# Comparison of four molecular approaches to identify *Candida parapsilosis* complex species

**DOI:** 10.1590/0074-02760160412

**Published:** 2017-02-16

**Authors:** Leonardo Silva Barbedo, Maria Helena Galdino Figueiredo-Carvalho, Mauro de Medeiros Muniz, Rosely Maria Zancopé-Oliveira

**Affiliations:** Fundação Oswaldo Cruz, Instituto Nacional de Infectologia Evandro Chagas, Laboratório de Micologia, Rio de Janeiro, RJ, Brasil

**Keywords:** *Candida parapsilosis* complex, Candida orthopsilosis, Candida metapsilosis, candidaemia, DNA, PCR

## Abstract

Since the description of *Candida orthopsilosis* and *C. metapsilosis* in 2005, several methods have been proposed to identify and differentiate these species from *C. parapsilosis* sensu stricto. Species-specific uniplex polymerase chain reaction (PCR) was performed and compared with sequencing of the D1/D2 region of the LSU 28S rDNA gene, microsatellite typing of *C. parapsilosis* sensu stricto, and PCR-restriction fragment length polymorphism patterns in the ITS1-5.8S-ITS2 region of the rDNA gene. There was agreement between results of testing of 98 clinical isolates with the four PCR-based methods, with 59 isolates identified as *C. parapsilosis* sensu stricto, 37 as *C. orthopsilosis*, and two as *C. metapsilosis*.

Candidaemia ranks as the third or fourth most common cause of health care-associated bloodstream infections, and it is a leading cause of hospitalisation of immunocompromised patients. Fungaemia caused by *Candida parapsilosis* is commonly associated with the provision of parenteral nutrition and use of a central venous catheter. *C. parapsilosis* is also the main species that causes bloodstream infections in premature newborns in neonatal intensive care units (Barbedo et al. 2016).

Since 1992, *C. parapsilosis* isolates have been recognised to form a complex that is phenotypically indistinguishable but genetically heterogeneous. The complex is composed of three distinct groups (groups I, II, and III) based on several criteria. Based on multilocus sequence typing (MLST) studies, Tavanti et al. (2005) proposed replacing the existing designations of *C. parapsilosis* groups II and III with the names *C. orthopsilosis* and *C. metapsilosis*, respectively, and suggested retaining the former designation *C. parapsilosis* for group I strains. Presently, *C. parapsilosis* sensu lato comprises *C. parapsilosis* sensu stricto and two newly recognised species.

Genotypic methods have advantages over phenotypic methods in the identification and characterisation of fungal isolates, mainly because of the stability of genomic markers and greater resolution in typing. Avni et al. (2011), in a systematic review and meta-analysis, showed that the pooled sensitivity and specificity of polymerase chain reaction (PCR)-based methods were 95% and 92%, respectively, in patients with suspected invasive candidiasis. Several DNA-based methods, such as direct PCR and sequencing with a BACTEC instrument (Pryce et al. 2006), randomly amplified polymorphic DNA (RAPD) analysis (Tay et al. 2009), restriction fragment length polymorphism (RFLP) pattern analysis (Lockhart et al. 2008, Silva et al. 2009, Mirhendi et al. 2010), amplified fragment length polymorphism (AFLP) analysis (Tavanti et al. 2007, Hensgens et al. 2009, de Carolis et al. 2014), real-time PCR (of the *SADH* gene and mitochondrial DNA) (Hays et al. 2011, Souza et al. 2012), intein sequence analysis (Prandini et al. 2013), uniplex and multiplex PCR (Asadzadeh et al. 2009, 2015), internal transcribed spacer 2 (ITS2) pyrosequencing (Borman et al. 2009), exon-primed intron-crossing (EPIC) PCR assay (Feng et al. 2014), *TOP2* loop-mediated isothermal amplification (LAMP) (Trabasso et al. 2015), and sequencing of specific regions (the D1/D2 domain, ITS1-5.8S-ITS2 and IGS1 regions of the rDNA gene, and RPS0 intron) (Gomez-Lopez et al. 2008, Vercher et al. 2011, Gago et al. 2014) have been used for the molecular identification and differentiation of *C. parapsilosis* complex species.

Although several molecular methods have been reported for identification and typing of species in the *C. parapsilosis* complex, as outlined above, there have been few comparisons of the results obtained using different molecular techniques with the same set of isolates. Therefore, the current work was carried out to compare four different molecular methods. The species-specific uniplex PCR was performed using specific primer pairs CPAF-CPAR, CORF-CORR, and CMEF-CMER, described by Asadzadeh et al. (2009), to discriminate between *C. parapsilosis* complex species and to compare to the partial D1/D2 region of LSU 28S rDNA gene sequences, PCR-RFLP patterns of the ITS1-5.8S-ITS2 region of the rDNA gene, and microsatellite typing of *C. parapsilosis* sensu stricto (Barbedo et al. 2015, 2016).

Ninety-eight clinical isolates of *C. parapsilosis* sensu lato obtained from the bloodstream and from catheter tips were included in this study. Information on all isolates, such as origin, biochemical characteristics, and DNA extraction, has been published previously (Barbedo et al. 2015, 2016). *C. parapsilosis* ATCC 22019, *C. orthopsilosis* ATCC 96141, and *C. metapsilosis* ATCC 96143 were used as reference strains.

All 98 clinical isolates were identified molecularly using species-specific primers. Fifty-nine isolates amplified with the primer pair CPAF-CPAR were identified as *C. parapsilosis* sensu stricto, 37 isolates amplified with primers CORF-CORR were identified as *C. orthopsilosis*, and two isolated amplified with primers CMEF-CMER were identified as *C. metapsilosis*. Figure illustrates the species-specific PCR of the reference strains and five representative isolates. These results agree with published sequences of the D1/D2 region of the LSU 28S rDNA gene, PCR-RFLP patterns of the ITS1-5.8S-ITS2 region of the rDNA gene, and microsatellite typing of *C. parapsilosis* sensu stricto (Barbedo et al. 2015, 2016) (Supplementary data, Table).

A protocol for species-specific uniplex PCR to rapidly discriminate between *C. parapsilosis* complex species using primers derived from unique sequences in the ITS1-5.8S-ITS2 region of the rDNA gene has been described. PCR amplification with primers CPAF-CPAR yielded an amplicon of 379 base pairs (bp) from DNA of *C. parapsilosis* sensu stricto isolates, whereas PCR amplification with CORF-CORR (*C. orthopsilosis)* and CMEF-CMER (*C. metapsilosis*) yielded amplicons of 367 bp and 374 bp, respectively, in agreement with results obtained by Asadzadeh et al. (2009). In summary, this methodology requires three different PCR reactions per sample. Despite the application of different pairs of primers, the annealing temperature used for all reactions was the same (63ºC), allowing the assays to be run at the same time in the same block of a thermal cycler. Although inaccuracies using this methodology has been reported, when compared with the *SADH* and *FKS1*-RFLP methods (Abi-Chacra et al. 2013), our results from the species-specific uniplex PCR were consistent with results from the other three techniques performed in this study.

The rDNA gene is the most common target for DNA-based detection methods in clinical and research laboratories for the following reasons: (i) several restriction sites for different enzymes are conserved in the rDNA of fungi; (ii) multiple copies of the ribosomal gene are present in all organisms, enabling sensitive detection by PCR; and (iii) the rDNA gene contains both highly conserved and variable regions, and is, therefore, the optimal target for specific PCR primers and restriction enzymes that discriminate among species. In the last 15 years, sequencing has been accepted as the gold standard for fungal identification and used to confirm the results of the other DNA-based identification methods. In general, PCR amplicons are sequenced to identify fungi at the species level using different DNA targets. PCR using primers that target highly variable regions within ITS1 and ITS2 and conserved regions of the 18S (small subunit, SSU), 5.8S, and 28S (large subunit, LSU) (e.g., D1/D2 domains) rDNA genes has been used to differentiate medically important *Candida* species (Jang et al. 2012).

There are several studies that use the first 600-900 bp of the LSU of the 28S rDNA gene, which comprises three divergent domains (D1, D2, and D3), to type *Candida* species. The D1/D2 domains were selected because of their high genetic variability, in contrast to the rest of the LSU, which is largely invariant across widely divergent taxa. In our study, sequences of the D1/D2 domains of the LSU of the 28S rDNA gene permitted discrimination between all *C. parapsilosis* complex species, and three clusters were generated, *C parapsilosis* sensu stricto, *C. orthopsilosis*, and *C. metapsilosis* (Barbedo et al. 2015) (Supplementary data, Fig. 1). The percentage similarity among the three *C. parapsilosis* complex species (ATCC strains) was 98.77% (563/570 bp). However, the point mutations present varied by species, so slight variation could be observed in our results. The percentage of similarities observed between species (the reference ATCC strains) in the *C. parapsilosis* complex were the following: 99.12% (565/570 bp) between *C. parapsilosis* and *C. orthopsilosis*, 98.95% (564/570 bp) between *C. parapsilosis* and *C. metapsilosis*, and 99.30% (566/570 bp) between *C. orthopsilosis* and *C. metapsilosis* (Supplementary data, Fig. 2).

Since the 1990s, RFLP has been used to type *Candida* species, and its use in conjunction with PCR has also been described. The majority of studies using RFLP analysis to differentiate between *C. parapsilosis* complex species have targeted partial *SADH* and *FKS1* genes (Table). In this study, PCR-RFLP of the ITS1-5.8S-ITS2 region of the rDNA gene was used to test the set of 98 isolates. Results were similar to those obtained with the other methods utilized. Double digestion with *Hha*I and *Sau96*I cut the ITS1-5.8S-ITS2 PCR products from these isolates in three ways, producing 117-, 178-, and 225-bp fragments for the 59 *C. parapsilosis* sensu stricto isolates; 102-, 183-, and 225-bp fragments for the 37 *C. orthopsilosis* isolates; and 114-, 187-, and 228-bp fragments for the two *C. metapsilosis* isolates (Barbedo et al. 2016).

**TABLE:** Summary of major studies published on the identification of *Candida parapsilosis* complex species by DNA-based methods

Reference	Source of specimens	Nº of isolates (%) by species			DNA-based methods used for differentiation

		*C. parapsilosis* s.s*.*	*C. orthopsilosis*	*C. metapsilosis*	
Tavanti et al. (2005)^*a*^	Different clinical sources	20 (74.1)	7 (25.9)	0 (0.0)	MLST; RAPD; *SADH*-RFLP
Pryce et al. (2006)	Blood and other clinical sources	8 (88.9)	1 (11.1)	0 (0.0)	Direct PCR-sequencing of BacTec
Tavanti et al. (2007)	Different clinical sources	277 (95.5)	13 (4.5)	0 (0.0)	*SADH*-RFLP; AFLP
Gomez-Lopez et al. (2008)	Blood	67 (85.9)	5 (6.4)	6 (7.7)	ITS sequencing
Lockhart et al. (2008)	Invasive clinical samples	1762 (92.1)	117 (6.1)	34 (1.8)	*SADH*-RFLP
Borman et al. (2009)	Different clinical sources	7 (33.3)	11 (52.4)	3 (14.3)	Pyrosequencing
Hensgens et al. (2009)	Different clinical sources	375 (94.9)	0 (0.0)	20 (5.1)	*SADH*-RFLP; AFLP
Tay et al. (2009)	Blood	29 (70.7)	10 (24.4)	2 (4.9)	ITS sequencing; RAPD
Asadzadeh et al. (2009)	Blood and other clinical sources	109 (95.6)	5 (4.4)	0 (0.0)	Species-specific PCR; *SADH*-RFLP; ITS and D1/D2 sequencing
Silva et al. (2009)	Clinical and environmental sources	160 (94.7)	4 (2.4)	5 (2.9)	*SADH*-RFLP
Mirhendi et al. (2010)	Blood	75 (95.0)	2 (2.5)	2 (2.5)	*SADH*-RFLP; *SADH*, ITS and D1/D2 sequencing
Hays et al. (2011)	Blood and other clinical sources	114 (98.3)	2 (1.7)	0 (0.0)	Real-time PCR; *SADH*-RFLP
Vercher et al. (2011)	Blood	30 (76.9)	5 (12.8)	4 (10.3)	RPS0 intron sequencing
Souza et al. (2012)	Different clinical sources	48 (50.0)	39 (40.6)	9 (9.4)	Real-time PCR
Prandini et al. (2013)	NI	46 (54.1)	22 (25.9)	17 (20.0)	Inteins sequencing
de Carolis et al. (2014)	Different clinical sources	12 (27.3)	19 (43.2)	13 (29.5)	AFLP
Feng et al*.* (2014)	Different clinical sources	112 (56.6)	30 (15.1)	56 (28.3)	Exon-primed intron-crossing PCR
Gago et al. (2014)	Different clinical sources	10 (33.3)	13 (43.4)	7 (23.3)	ITS sequencing; High-resolution melting analysis
Trabasso et al. (2015)	Blood	31 (86.1)	4 (11.1)	1 (2.8)	ITS sequencing; *TOP2*-LAMP
Asadzadeh et al. (2015)	Blood	361 (95.7)	15 (3.6)	1 (0.3)	Multiplex PCR; AFLP; ITS and D1/D2 sequencing

*a*: studies realised after Tavanti et al. (2005); NI: no information; s.s.: sensu stricto.

Microsatellites are defined as short 2-bp to 10-bp tandem repeats, and they are increasingly being used as genetic markers. This methodology has an advantage over other typing methods. Microsatellites behave as codominant markers, evolve rapidly in the genome, and permit detection of microevolutionary variation that allows isolates to be distinguished from one another. In addition, microsatellites are the markers most commonly used for differentiation of isolates because of their hypervariability, ease of PCR amplification and interpretation, and their potential for use in automated assays to determine routes of transmission, strain persistence, and outbreak or relapse sources. Microsatellites have successfully been used to type several yeast species, including *Saccharomyces cerevisiae*, *C. albicans*, *C. krusei*, and *C. glabrata* (Lasker et al. 2006).

The first method that enabled isolates within the *C. parapsilosis* sensu stricto group to be distinguished was a microsatellite method based on dinucleotide repeats described by Lasker et al. (2006). However, the combined discriminatory power (DP) of 0.97 achieved with all seven loci (six dinucleotide and one trinucleotide) was not optimal. Furthermore, the typing of dinucleotide microsatellites limits accurate allele identification, even in automatic systems, because of the high frequency of dinucleotide repeat slippage. A new DNA-typing tool using microsatellite length polymorphisms that enabled the subtyping of *C. parapsilosis* sensu stricto was described in 2010 (three trinucleotide microsatellites: CP1, CP4, and CP6) and modified in 2001 (microsatellite CP4a). It presented a combined DP of 0.99. Because this multiplex strategy is specific to *C. parapsilosis* sensu stricto, four microsatellites (CP1, CP4a, CP6, and B) were targeted in the 98 clinical isolates. The multiplex microsatellite PCR failed to amplify all markers in 39 clinical isolates (*C. orthopsilosis* and *C. metapsilosis* isolates); thus, only 59 *C. parapsilosis* sensu stricto clinical isolates were amplified (identifying 39 multilocus genotypes) with this method (Barbedo et al. 2015). The failure to amplify these microsatellite markers (CP1, CP4a, CP6, and B) appeared to be restricted to *C. orthopsilosis* or *C. metapsilosis* isolates.

Recently, Jia (2016) analysed microsatellite and compound microsatellite distribution, composition, and polymorphisms in three *Candida* genomes. The results showed that there were 118,047; 66,259; and 61,119 microsatellites in the genomes of *C. dubliniensis*, *C. glabrata*, and *C. orthopsilosis*, respectively. The microsatellites covered more than one-third the length of the genomes of the three species, and consisted only of bases A and/or T, such as mononucleotides (A)n and (T)n; dinucleotides (AT)n and (TA)n; and trinucleotides (AAT)n, (TAA)n, (TTA)n, (ATA)n, (ATT)n, and (TAT)n that were predominant in the three genomes. This analysis may be useful for further studies on the roles of repeat sequences in *Candida* species evolution and organisation.

Table presents the different DNA-based techniques used for genetic differentiation of *C. parapsilosis* complex species. In the majority of studies (except for three), the proportion of isolates that were *C. parapsilosis* sensu stricto was greater than that of *C. orthopsilosis*, whereas *C. orthopsilosis* was more frequently detected than *C. metapsilosis* (except in four studies), in agreement with our findings. The majority of studies that compared results obtained from different DNA-based methods used two to four methodologies. However, this is the first time that specie-specific uniplex PCR, D1/D2 sequencing, microsatellite typing of *C. parapsilosis* sensu stricto, and PCR-RFLP of the ITS1-5.8S-ITS2 region of the rDNA gene have been compared using the same set of organisms. The ITS1-5.8S-ITS2 region and D1/D2 domains of the LSU of the 28S rDNA gene were the main regions targeted for sequencing. Curiously, in a study by Mirhendi et al. (2010), *SADH*-RFLP with *Ban*I did not allow identification of *C. metapsilosis*. The enzyme *Ban*I successfully distinguished between *C. parapsilosis* sensu stricto and two other species, but the band profiles of *C. orthopsilosis* and *C. metapsilosis* were so similar that misclassification of these two species should be expected in routine testing.

The *C. parapsilosis* complex is highly heterogeneous. Some studies have indicated that clinical *C. orthopsilosis* isolates are more genetically diverse than *C. parapsilosis* sensu stricto isolates, which are predominantly clonal and exhibit limited genotypic variation. According to Tay et al. (2009), based on sequencing of ITS region, and Sai et al. (2011), based on *MTL* (mating-type) loci, *C. orthopsilosis* can be divided into at least two subspecies (type 1 and type 2). In addition, Asadzadeh et al. (2015) identified three different haplotypes among 19 *C. orthopsilosis* isolates, based on nine divergent nucleotides in the ITS region.

The genome of a *C. orthopsilosis* type 2 isolate was sequenced in 2012, which was useful for extensive comparison with the genome of *C. parapsilosis* sensu stricto. The main differences were an expansion of the Hyr/Iff family of cell wall genes and the JEN family of monocarboxylic transporters in *C. parapsilosis* sensu stricto, relative to those in *C. orthopsilosis*. More recently, Pryszcz et al. (2014) sequenced *C. orthopsilosis* type 1 and compared the sequence to those of other strains, because they believed that the lack of sequences from additional strains, particularly from other subspecies, has limited understanding of the genomic variability within this species. The results of their study indicated that the newly sequenced isolate likely represented a hybrid between the two *C. orthopsilosis* subspecies. The hybrid lineage is represented by at least two clinical isolates from distant continents, suggesting its global spread. In addition, they found no evidence of an increase in ploidy or meiotic recombination between the different haplotypes, strongly suggesting mating between strains from different *C. orthopsilosis* lineages. In addition, several differences in the sequences of virulence-related gene families in the two strains were observed, including a larger copy number of efflux pumps and secreted lipases in the hybrid. These findings raise the question of what role *Candida* hybrids might play in the success and spread of the pathogen (Pryszcz et al. 2014).

The diagnosis of candidiasis based on molecular techniques has become increasingly important, particularly for research on nosocomial candidiasis in patients at increased risk, as well as studies on the diversity and dynamics of *Candida* species. Current gold standards for the diagnosis of invasive fungal infections by serologic and phenotypic methods lack sensitivity and speed, resulting in delayed treatment and decreased survival of candidiasis patients. Although novel methods for the detection and identification of fungal pathogens have been developed, inconsistencies between different approaches limit the reproducibility of results and prohibit large-scale clinical implementation. In contrast, the sensitivity of molecular methods raises the possibility of identifying infections at a very early stage, when it is easier to treat or even prevent clinical manifestations. PCR is one of the oldest and most widely used molecular fungal diagnostic methods, and in some cases of invasive candidiasis with discordant results between blood cultures and PCR, *Candida* species were isolated from other sterile sites in the patients, indicating that the PCR result was a true positive and suggesting the superiority of this assay over the traditional gold standard diagnostic test (Arvanitis et al. 2014).

In this study, results obtained from the four PCR-based methods were 100% concordant for our 98 clinical isolates. The methods used were (i) species-specific uniplex PCR, (ii) sequencing of the D1/D2 region of the LSU 28S rDNA gene, (iii) PCR-RFLP analysis, and (iv) microsatellite typing of *C. parapsilosis* sensu stricto. The agreement in results indicates that the four methods are all reliable and feasible. Therefore, because all four methodologies distinguished between species in the *C. parapsilosis* complex, they could all theoretically be chosen for identification. However, important parameters such as cost, complexity, and expertise needed to perform each assay also need to be considered, i.e., species-specific uniplex PCR and PCR-RFLP methods should be considered in laboratories with limited financial resources.

Sequencing of the D1/D2 region of the LSU 28S rDNA gene enabled identification of *C. parapsilosis* complex species with the detection of point mutations, even though this region has seven divergent nucleotides. However, this method is time consuming and very expensive for use in the routine diagnosis of candidiasis. On the other hand, microsatellites can be used to distinguish between and type *C. parapsilosis* sensu stricto strains, and therefore are valuable for epidemiological studies of related isolates, including nosocomially-transmitted strains, as well as studies on the kinetics of colonization-to-infection. Identifying microsatellites in *C. metapsilosis* and those recently identified in the *C. orthopsilosis* genome may be helpful for similar studies.

In conclusion, the methodologies described in this work can be used for identification and differentiation of *C. parapsilosis* complex species and for typing of *C. parapsilosis* sensu stricto strains and are important tools for use in outbreaks and epidemiological investigations.

**Figure:**
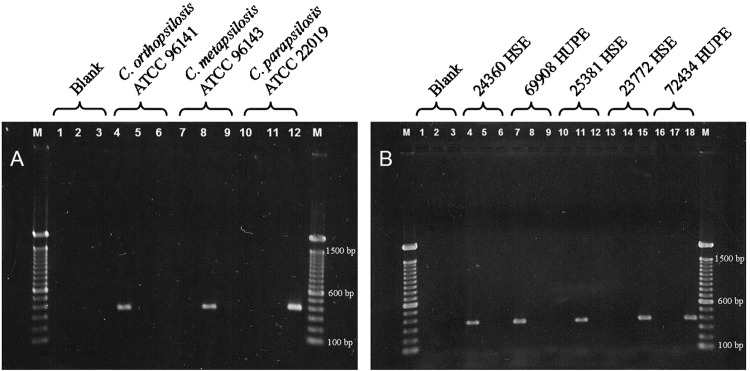
Electrophoresis of species-specific polymerase chain reaction (PCR) products. (A) American Type Culture Collection reference strains amplified with primers CORF-CORR (lanes 1, 4, 7, and 10), CMER-CMER (lanes 2, 5, 8, and 11), and CPAF-CPAR (lanes 3, 6, 9, and 12); (B) five representative clinical isolates amplified with primers CORF-CORR (lanes 1, 4, 7, 10, 13, and 16), CMER-CMER (lanes 2, 5, 8, 11, 14, and 17), and CPAF-CPAR (lanes 3, 6, 9, 12, 15, and 18). Isolates 24360 Hospital dos Servidores do Estado (HSE) and 69908 Hospital Universitário Pedro Ernesto (HUPE) were identified as *Candida orthopsilosis*, whilst isolate 25381 HSE was identified as *C. metapsilosis* and isolates 23772 HSE and 72434 HUPE were identified as *C. parapsilosis sensu stricto*. Lane M, 100-bp DNA ladder (marker).
