# Supplementary material for: Comparison of four molecular approaches to identify Candida parapsilosis complex species
Source: Mem Inst Oswaldo Cruz. 2017 Feb 16;112(3):214–9. doi: 10.1590/0074-02760160412 (PMC5319372; doi:10.1590/0074-02760160412)
Supplement: Supplementary file 1 [file 0074-0276-mioc-0074-02760160412-suppl01.pdf]

TABLE

Results of all clinical isolates analysed in this study. Species-specific polymerase chain reaction (PCR) (this work); sequences of D1/D2 domains and microsatellites (Barbedo et al. 2015), and PCR-restriction fragment length polymorphism (PCR-RFLP) (Barbedo et al. 2016)

Isolate	Sample	Hospital	Origin	Species-specific PCR	D1/D2 domains	Microsatellites	PCR-RFLP
1	23772	HSE	Blood	*C. parapsilosis*	*C. parapsilosis*	*C. parapsilosis*	*C. parapsilosis*
2	23795	HSE	Blood	*C. parapsilosis*	*C. parapsilosis*	*C. parapsilosis*	*C. parapsilosis*
3	24258	HSE	Blood	*C. parapsilosis*	*C. parapsilosis*	*C. parapsilosis*	*C. parapsilosis*
4	24360	HSE	Blood	*C. orthopsilosis*	*C. orthopsilosis*	NA	*C. orthopsilosis*
5	24384	HSE	Blood	*C. parapsilosis*	*C. parapsilosis*	*C. parapsilosis*	*C. parapsilosis*
6	24775	HSE	Blood	*C. orthopsilosis*	*C. orthopsilosis*	NA	*C. orthopsilosis*
7	32485	HSE	Blood	*C. parapsilosis*	*C. parapsilosis*	*C. parapsilosis*	*C. parapsilosis*
8	32504	HSE	Blood	*C. parapsilosis*	*C. parapsilosis*	*C. parapsilosis*	*C. parapsilosis*
9	33286	HSE	Blood	*C. parapsilosis*	*C. parapsilosis*	*C. parapsilosis*	*C. parapsilosis*
10	33499	HSE	Blood	*C. parapsilosis*	*C. parapsilosis*	*C. parapsilosis*	*C. parapsilosis*
11	33524	HSE	Blood	*C. parapsilosis*	*C. parapsilosis*	*C. parapsilosis*	*C. parapsilosis*
12	71663	HSE	Catheter	*C. parapsilosis*	*C. parapsilosis*	*C. parapsilosis*	*C. parapsilosis*
13	72102	HSE	Catheter	*C. parapsilosis*	*C. parapsilosis*	*C. parapsilosis*	*C. parapsilosis*
14	72737	HSE	Catheter	*C. parapsilosis*	*C. parapsilosis*	*C. parapsilosis*	*C. parapsilosis*
15	72834	HSE	Catheter	*C. parapsilosis*	*C. parapsilosis*	*C. parapsilosis*	*C. parapsilosis*
16	72836	HSE	Catheter	*C. orthopsilosis*	*C. orthopsilosis*	NA	*C. orthopsilosis*
17	73234	HSE	Catheter	*C. orthopsilosis*	*C. orthopsilosis*	NA	*C. orthopsilosis*
18	75234	HSE	Catheter	*C. orthopsilosis*	*C. orthopsilosis*	NA	*C. orthopsilosis*
19	75525	HSE	Catheter	*C. parapsilosis*	*C. parapsilosis*	*C. parapsilosis*	*C. parapsilosis*
20	76542	HSE	Catheter	*C. parapsilosis*	*C. parapsilosis*	*C. parapsilosis*	*C. parapsilosis*
21	76555	HSE	Catheter	*C. parapsilosis*	*C. parapsilosis*	*C. parapsilosis*	*C. parapsilosis*
22	76883	HSE	Catheter	*C. parapsilosis*	*C. parapsilosis*	*C. parapsilosis*	*C. parapsilosis*
23	78981	HSE	Catheter	*C. parapsilosis*	*C. parapsilosis*	*C. parapsilosis*	*C. parapsilosis*
24	79130	HSE	Catheter	*C. parapsilosis*	*C. parapsilosis*	*C. parapsilosis*	*C. parapsilosis*
25	79182	HSE	Catheter	*C. parapsilosis*	*C. parapsilosis*	*C. parapsilosis*	*C. parapsilosis*
26	79431	HSE	Catheter	*C. parapsilosis*	*C. parapsilosis*	*C. parapsilosis*	*C. parapsilosis*
27	79769	HSE	Catheter	*C. parapsilosis*	*C. parapsilosis*	*C. parapsilosis*	*C. parapsilosis*
28	ROM001	HSE	Blood	*C. metapsilosis*	*C. metapsilosis*	NA	*C. metapsilosis*
29	2500096400	SAM	Blood	*C. parapsilosis*	*C. parapsilosis*	*C. parapsilosis*	*C. parapsilosis*
30	2500100450	SAM	Blood	*C. parapsilosis*	*C. parapsilosis*	*C. parapsilosis*	*C. parapsilosis*
31	2500100454	SAM	Catheter	*C. parapsilosis*	*C. parapsilosis*	*C. parapsilosis*	*C. parapsilosis*
32	25011112911	SAM	Blood	*C. parapsilosis*	*C. parapsilosis*	*C. parapsilosis*	*C. parapsilosis*
33	69908	HUPE	Blood	*C. orthopsilosis*	*C. orthopsilosis*	NA	*C. orthopsilosis*
34	70114	HUPE	Blood	*C. orthopsilosis*	*C. orthopsilosis*	NA	*C. orthopsilosis*
35	70158	HUPE	Blood	*C. parapsilosis*	*C. parapsilosis*	*C. parapsilosis*	*C. parapsilosis*
36	70186	HUPE	Blood	*C. orthopsilosis*	*C. orthopsilosis*	NA	*C. orthopsilosis*
37	71228	HUPE	Blood	*C. orthopsilosis*	*C. orthopsilosis*	NA	*C. orthopsilosis*
38	72434	HUPE	Blood	*C. parapsilosis*	*C. parapsilosis*	*C. parapsilosis*	*C. parapsilosis*
39	72728	HUPE	Blood	*C. parapsilosis*	*C. parapsilosis*	*C. parapsilosis*	*C. parapsilosis*
40	72820	HUPE	Blood	*C. parapsilosis*	*C. parapsilosis*	*C. parapsilosis*	*C. parapsilosis*
41	72864	HUPE	Blood	*C. parapsilosis*	*C. parapsilosis*	*C. parapsilosis*	*C. parapsilosis*
42	73172	HUPE	Blood	*C. parapsilosis*	*C. parapsilosis*	*C. parapsilosis*	*C. parapsilosis*
43	73204	HUPE	Blood	*C. parapsilosis*	*C. parapsilosis*	*C. parapsilosis*	*C. parapsilosis*
44	79074	HUPE	Blood	*C. parapsilosis*	*C. parapsilosis*	*C. parapsilosis*	*C. parapsilosis*
45	79188	HUPE	Blood	*C. orthopsilosis*	*C. orthopsilosis*	NA	*C. orthopsilosis*
46	79298 B	HUPE	Blood	*C. orthopsilosis*	*C. orthopsilosis*	NA	*C. orthopsilosis*
47	80550	HUPE	Blood	*C. orthopsilosis*	*C. orthopsilosis*	NA	*C. orthopsilosis*
48	80792	HUPE	Blood	*C. parapsilosis*	*C. parapsilosis*	*C. parapsilosis*	*C. parapsilosis*

Isolate	Sample	Hospital	Origin	Species-specific PCR	D1/D2 domains	Microsatellites	PCR-RFLP
49	80828	HUPE	Blood	*C. parapsilosis*	*C. parapsilosis*	*C. parapsilosis*	*C. parapsilosis*
50	80846	HUPE	Blood	*C. parapsilosis*	*C. parapsilosis*	*C. parapsilosis*	*C. parapsilosis*
51	80882	HUPE	Blood	*C. parapsilosis*	*C. parapsilosis*	*C. parapsilosis*	*C. parapsilosis*
52	81480	HUPE	Blood	*C. orthopsilosis*	*C. orthopsilosis*	NA	*C. orthopsilosis*
53	22988	HSE	Blood	*C. orthopsilosis*	*C. orthopsilosis*	NA	*C. orthopsilosis*
54	23027	HSE	Blood	*C. parapsilosis*	*C. parapsilosis*	*C. parapsilosis*	*C. parapsilosis*
55	23048	HSE	Blood	*C. parapsilosis*	*C. parapsilosis*	*C. parapsilosis*	*C. parapsilosis*
56	23175	HSE	Blood	*C. orthopsilosis*	*C. orthopsilosis*	NA	*C. orthopsilosis*
57	23637	HSE	Blood	*C. parapsilosis*	*C. parapsilosis*	*C. parapsilosis*	*C. parapsilosis*
58	23657	HSE	Blood	*C. parapsilosis*	*C. parapsilosis*	*C. parapsilosis*	*C. parapsilosis*
59	24213	HSE	Blood	*C. orthopsilosis*	*C. orthopsilosis*	NA	*C. orthopsilosis*
60	24323	HSE	Blood	*C. orthopsilosis*	*C. orthopsilosis*	NA	*C. orthopsilosis*
61	24391	HSE	Blood	*C. parapsilosis*	*C. parapsilosis*	*C. parapsilosis*	*C. parapsilosis*
62	24412	HSE	Blood	*C. orthopsilosis*	*C. orthopsilosis*	NA	*C. orthopsilosis*
63	24853	HSE	Blood	*C. parapsilosis*	*C. parapsilosis*	*C. parapsilosis*	*C. parapsilosis*
64	25381	HSE	Blood	*C. metapsilosis*	*C. metapsilosis*	NA	*C. metapsilosis*
65	26451	HSE	Blood	*C. orthopsilosis*	*C. orthopsilosis*	NA	*C. orthopsilosis*
66	26514(1)	HSE	Blood	*C. orthopsilosis*	*C. orthopsilosis*	NA	*C. orthopsilosis*
67	26860	HSE	Blood	*C. orthopsilosis*	*C. orthopsilosis*	NA	*C. orthopsilosis*
68	26860 R	HSE	Blood	*C. orthopsilosis*	*C. orthopsilosis*	NA	*C. orthopsilosis*
69	26874	HSE	Blood	*C. parapsilosis*	*C. parapsilosis*	*C. parapsilosis*	*C. parapsilosis*
70	26882	HSE	Blood	*C. parapsilosis*	*C. parapsilosis*	*C. parapsilosis*	*C. parapsilosis*
71	26906	HSE	Blood	*C. orthopsilosis*	*C. orthopsilosis*	NA	*C. orthopsilosis*
72	26981(1)	HSE	Blood	*C. orthopsilosis*	*C. orthopsilosis*	NA	*C. orthopsilosis*
73	26989	HSE	Blood	*C. parapsilosis*	*C. parapsilosis*	*C. parapsilosis*	*C. parapsilosis*
74	26990(1)	HSE	Blood	*C. orthopsilosis*	*C. orthopsilosis*	NA	*C. orthopsilosis*
75	27246	HSE	Blood	*C. orthopsilosis*	*C. orthopsilosis*	NA	*C. orthopsilosis*
76	27401	HSE	Blood	*C. parapsilosis*	*C. parapsilosis*	*C. parapsilosis*	*C. parapsilosis*
77	27515	HSE	Blood	*C. parapsilosis*	*C. parapsilosis*	*C. parapsilosis*	*C. parapsilosis*
78	27956	HSE	Blood	*C. orthopsilosis*	*C. orthopsilosis*	NA	*C. orthopsilosis*
79	27969	HSE	Blood	*C. orthopsilosis*	*C. orthopsilosis*	NA	*C. orthopsilosis*
80	28118	HSE	Blood	*C. parapsilosis*	*C. parapsilosis*	*C. parapsilosis*	*C. parapsilosis*
81	28241	HSE	Blood	*C. parapsilosis*	*C. parapsilosis*	*C. parapsilosis*	*C. parapsilosis*
82	28243	HSE	Blood	*C. parapsilosis*	*C. parapsilosis*	*C. parapsilosis*	*C. parapsilosis*
85	28684	HSE	Blood	*C. parapsilosis*	*C. parapsilosis*	*C. parapsilosis*	*C. parapsilosis*
86	28947	HSE	Blood	*C. orthopsilosis*	*C. orthopsilosis*	NA	*C. orthopsilosis*
87	29176	HSE	Blood	*C. orthopsilosis*	*C. orthopsilosis*	NA	*C. orthopsilosis*
88	29936	HSE	Blood	*C. parapsilosis*	*C. parapsilosis*	*C. parapsilosis*	*C. parapsilosis*
89	29954(1)	HSE	Blood	*C. orthopsilosis*	*C. orthopsilosis*	NA	*C. orthopsilosis*
90	30842	HSE	Blood	*C. orthopsilosis*	*C. orthopsilosis*	NA	*C. orthopsilosis*
91	31949	HSE	Blood	*C. orthopsilosis*	*C. orthopsilosis*	NA	*C. orthopsilosis*
92	32095(1)	HSE	Blood	*C. orthopsilosis*	*C. orthopsilosis*	NA	*C. orthopsilosis*
93	32095(2)	HSE	Blood	*C. parapsilosis*	*C. parapsilosis*	*C. parapsilosis*	*C. parapsilosis*
94	32136	HSE	Blood	*C. orthopsilosis*	*C. orthopsilosis*	NA	*C. orthopsilosis*
95	32804	HSE	Blood	*C. parapsilosis*	*C. parapsilosis*	*C. parapsilosis*	*C. parapsilosis*
96	32889	HSE	Blood	*C. orthopsilosis*	*C. orthopsilosis*	NA	*C. orthopsilosis*
97	32988	HSE	Blood	*C. orthopsilosis*	*C. orthopsilosis*	NA	*C. orthopsilosis*
98	33044	HSE	Blood	*C. parapsilosis*	*C. parapsilosis*	*C. parapsilosis*	*C. parapsilosis*
99	34094	HSE	Blood	*C. parapsilosis*	*C. parapsilosis*	*C. parapsilosis*	*C. parapsilosis*
100	79738	HSE	Blood	*C. parapsilosis*	*C. parapsilosis*	*C. parapsilosis*	*C. parapsilosis*

HSE: Hospital dos Servidores do Estado; HUPE: Hospital Universitário Pedro Ernesto; SAM: Hospital Samaritano; NA: no amplification.

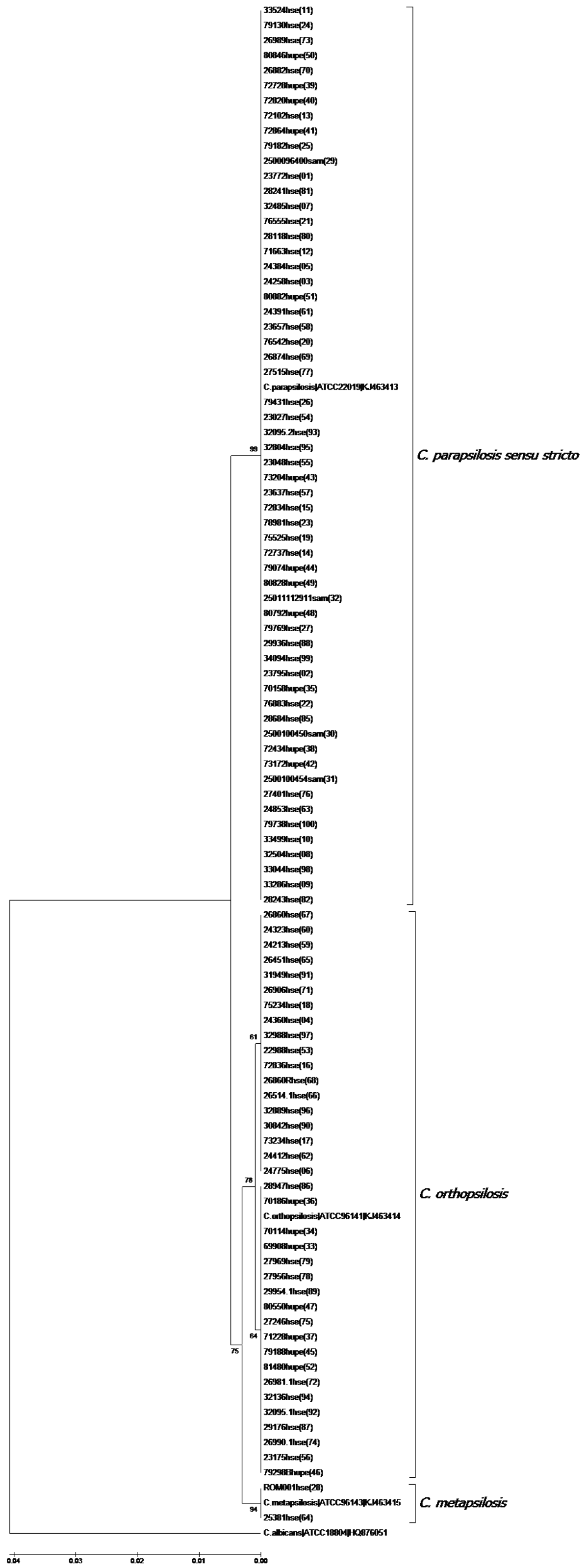

Fig. 1: evolutionary relationships of 102 taxa based on analysis of D1/D2 region of the LSU 28S rDNA gene. Ninety-eight clinical isolates and four ATCC references strains from NCBI/GenBank. The evolutionary history was inferred using the UPGMA method. The percentage of replicate trees in which the associated taxa clustered together in the bootstrap test (1000 replicates) are shown next to the branches. The evolutionary distances were computed using the Maximum Composite Likelihood method and are in the units of the number of base substitutions per site. There were a total of 575 positions in the final dataset. Evolutionary analyses were conducted in MEGA 6.06 software. HSE: Hospital dos Servidores do Estado; HUPE: Hospital Universitário Pedro Ernesto; SAM: Hospital Samaritano.

```
C.parapsilosis|ATCC22019      AAACCAACAGGGATTGCCTTAGTAGCGGCGAGTGAAGCGGCAAAAGCTCA 50
C.orthopsilosis|ATCC96141     AAACCAACAGGGATTGCCTTAGTAGCGGCGAGTGAAGCGGCAAAAGCTCA 50
C.metapsilosis|ATCC96143      AAACCAACAGGGATTGCCTTAGTAGCGGCGAGTGAAGCGGCAAAAGCTCA 50
                              **************************************************

C.parapsilosis|ATCC22019      AATTTGAAATCTGGCACTTTCAGTGTCCGAGTTGTAATTTGAAGAAGGTA 100
C.orthopsilosis|ATCC96141     AATTTGAAATCTGGCACTTTCAGTGTCCGAGTTGTAATTTGAAGAAGGTA 100
C.metapsilosis|ATCC96143      AATTTGAAATCTGGCACTTTCAGTGTCCGAGTTGTAATTTGAAGAAGGTA 100
                              **************************************************

C.parapsilosis|ATCC22019      TCTTTGGGTCTGGCTCTTGTCTATGTTTCTTGGAACAGAACGTCACAGAG 150
C.orthopsilosis|ATCC96141     TCTTTGGGTCTGGCTCTTGTCTATGTTTCTTGGAACAGAACGTCACAGAG 150
C.metapsilosis|ATCC96143      TCTTTGGGTCTGGCTCTTGTCTATGTTTCTTGGAACAGAACGTCACAGAG 150
                              **************************************************

C.parapsilosis|ATCC22019      GGTGAGAATCCCGTGCGATGAGATGTCCCAGACCTATGTAAAGTTCCTTC 200
C.orthopsilosis|ATCC96141     GGTGAGAATCCCGTGCGATGAGATGTCCCAGACCTATGTAAAGTTCCTTC 200
C.metapsilosis|ATCC96143      GGTGAGAATCCCGTGCGATGAGATGACCCAGACCTATGTAAAGTTCCTTC 200
                              ************************* ************************

C.parapsilosis|ATCC22019      GAAGAGTCGAGTTGTTTGGGAATGCAGCTCTAAGTGGGTGGTAAATTCCA 250
C.orthopsilosis|ATCC96141     GAAGAGTCGAGTTGTTTGGGAATGCAGCTCTAAGTGGGTGGTAAATTCCA 250
C.metapsilosis|ATCC96143      GAAGAGTCGAGTTGTTTGGGAATGCAGCTCTAAGTGGGTGGTAAATTCCA 250
                              **************************************************

C.parapsilosis|ATCC22019      TCTAAAGCTAAATATTGGCGAGAGACCGATAGCGAACAAGTACAGTGATG 300
C.orthopsilosis|ATCC96141     TCTAAAGCTAAATATTGGCGAGAGACCGATAGCGAACAAGTACAGTGATG 300
C.metapsilosis|ATCC96143      TCTAAAGCTAAATATTGGCGAGAGACCGATAGCGAACAAGTACAGTGATG 300
                              **************************************************

C.parapsilosis|ATCC22019      GAAAGATGAAAAGAACTTTGAAAAGAGAGTGAAAAAGTACGTGAAATTGT 350
C.orthopsilosis|ATCC96141     GAAAGATGAAAAGAACTTTGAAAAGAGAGTGAAAAAGTACGTGAAATTGT 350
C.metapsilosis|ATCC96143      GAAAGATGAAAAGAACTTTGAAAAGAGAGTGAAAAAGTACGTGAAATTGT 350
                              **************************************************

C.parapsilosis|ATCC22019      TGAAAGGGAAGGGCTTGAGATCAGACTTGGTATTTTGTATGTTACTCTCT 400
C.orthopsilosis|ATCC96141     TGAAAGGGAAGGGCTTGAGATCAGACTTGGTATTTTGTATGTTACTCTCT 400
C.metapsilosis|ATCC96143      TGAAAGGGAAGGGCTTGAGATCAGACTTGGTATTTTGTATGTTACTCTTT 400
                              ************************************************ *

C.parapsilosis|ATCC22019      CGGGGGTGGCCTCTACAGTTTACCGGGCCAGCATCAGTTTGAGCGGTAGG 450
C.orthopsilosis|ATCC96141     CGGGGGTGGCCTCTACAGTTTACCGGGCCAGCATCAGTTTGGGCGGTAGG 450
C.metapsilosis|ATCC96143      CGGGGGTGGCCTCTACAGTTTACCGGGCCAGCATCAGTTTGGGCGGTAGG 450
                              ***************************************** ********

C.parapsilosis|ATCC22019      ATAAGTGCAAAGAAATGTGGCACTGCTTCGGTAGTGTGTTATAGTCTTTG 500
C.orthopsilosis|ATCC96141     ACAATTGCAAAGAAATGTGGCACTGCCTCGGTAGTGTGTTATAGTCTTTG 500
C.metapsilosis|ATCC96143      AGAATTGCAAAGAAATGTGGCACTGCTTCGGTAGTGTGTTATAGTCTTTG 500
                              * ** ********************* ***********************

C.parapsilosis|ATCC22019      TCGATACTGCCAGCTTAGACTGAGGACTGCGGCTTCGGCCTAGGATGTTG 550
C.orthopsilosis|ATCC96141     TCGATACTGCCAGCCTAGACTGAGGACTGCGGCTTCGGCCTAGGATGTTG 550
C.metapsilosis|ATCC96143      TCGATACTGCCAGCCTAGACTGAGGACTGCGGCTTCGGCCTAGGATGTTG 550
                              ************** ***********************************

C.parapsilosis|ATCC22019      GCATAATGATCTTAAGTCGC 570
C.orthopsilosis|ATCC96141     GCATAATGATCTTAAGTCGC 570
C.metapsilosis|ATCC96143      GCATAATGATCTTAAGTCGC 570
                              ********************
```

Fig. 2: alignment of the D1\D2 region of the LSU 28S rDNA gene of the *C. parapsilosis* complex species, *C. parapsilosis* ATCC 22019, *C. orthopsilosis* ATCC 96141 and *C. metapsilosis* ATCC 96143 from GenBank under accession numbers KJ463413, KJ463414 and KJ463415, respectively. Nucleotides that are identical in all three species are indicated by an asterisk below the sequence. This alignment does not include primers (NL-1 and NL-4) regions. There are seven divergent nucleotides positions: 176, 399, 442, 452, 455, 477 and 515. Alignment made with Clustal Omega tool (http://www.ebi.ac.uk/Tools/msa/clustalo/).

Supplementary data
